# MTSET modification of D4S6 cysteines stabilize the fast inactivated state of Na_v_1.5 sodium channels

**DOI:** 10.3389/fphar.2015.00118

**Published:** 2015-06-19

**Authors:** Michael E. O’Leary, Mohamed Chahine

**Affiliations:** ^1^Department of Biomedical Sciences, Cooper Medical School of Rowan University, Camden, NJUSA; ^2^Department of Medicine, Research Centre, Institute Universitaire en Santé Mentale de Québec, Laval UniversityQuébec, QC, Canada

**Keywords:** sodium channel, Nav1.5, D4S6, MTSET, DIVS6, Y1767, F1760, V1763

## Abstract

The transmembrane S6 segments of Na^+^ sodium channels form the cytoplasmic entrance of the channel and line the internal aspects of the aqueous pore. This region of the channel has been implicated in Na^+^ channel permeation, gating, and pharmacology. In this study we utilized cysteine substitutions and methanethiosulfonate reagent (MTSET) to investigate the role of the S6 segment of homologous domain 4 (D4S6) in the gating of the cardiac (Na_v_1.5) channel. D4S6 cysteine mutants were heterologously expressed in tsA201 cells and currents recorded using whole-cell patch clamp. Internal MTSET reduced the peak Na^+^ currents, induced hyperpolarizing shifts in steady-state inactivation and slowed the recovery of mutant channels with cysteines inserted near the middle (F1760C, V1763C) and C-terminus (Y1767C) of the D4S6. These findings suggested a link between the MTSET inhibition and fast inactivation. This was confirmed by expressing the V1763C and Y1767C mutations in non-inactivating Na_v_1.5 channels. Removing inactivation abolished the MTSET inhibition of the V1763C and Y1767C mutants. The data indicate that the MTSET-induced reduction in current primarily results from slower recovery from inactivation that produces hyperpolarizing shifts in fast inactivation and decreases the steady-state availability of the channels. This contrasted with a cysteine inserted near the C-terminus of the D4S6 (I1770C) where MTSET increased the persistent Na^+^ current at depolarized voltages consistent with impaired fast inactivation. Covalent modification of D4S6 cysteines with MTSET adduct appears to reduce the mobility of the D4S6 segment and stabilize the channels in the fast inactivated state. These findings indicate that residues located near the middle and C-terminus of the D4S6 play an important role in fast inactivation.

## Introduction

Voltage-gated Na_v_1.5 Na^+^ channels produce the rapid upstroke of the cardiac action potential that serves as a trigger for the downstream events leading to myocardial contraction. These channels are important determinants of myocardium excitability, electrical conduction and are the target of class I antiarrhythmic drugs (Nau et al., 1999; Lipkind and Fozzard, 2005; Ulbricht, 2005; Catterall and Swanson, 2015). Naturally occurring mutations of Na_v_1.5 cause hereditary cardiac arrhythmias including Long QT type-3 (LQTS3) and Brugada syndromes (Huang et al., 2006; Millat et al., 2009). The Na_v_1.5 channel α subunit is organized into four homologous domains (D1–D4) each composed of six transmembrane segments (S1–S6). The α subunits encode for the basic properties of the channel including ion permeation and voltage-dependent gating (Catterall, 2000, 2012). The S6 segments contributed by the four homologous domains (D1S6–D4S6) of Na_v_1.5 converge to form the cytoplasmic entrance of channel (Payandeh et al., 2011; Bagneris et al., 2015). The cytoplasmic pore region includes residues that contribute to ion permeation, channel gating and the binding of pore-blocking drugs (Sheets et al., 2010; Catterall and Swanson, 2015).

Residues of the D4S6 segment are proposed to line the cytoplasmic pore of Na^+^ channels. The exposure of specific D4S6 residues within the aqueous pore is supported by the crystal structures of bacterial Na^+^ channels (Payandeh et al., 2011; Bagneris et al., 2015). In this study we employed a combination of mutagenesis and cysteine-specific modifying reagents to investigate the role of the D4S6 in Na^+^ channel gating. The data show that MTSET modification of cysteines exposed within the aqueous pore slows recovery from inactivation, produces hyperpolarizing shifts in steady-state availability, and reduces Na^+^ current amplitude. MTSET inhibition of the D4S6 mutant Na^+^ currents was abolished by mutations of the interdomain D3–D4 linker that remove fast inactivation. MTSET modification of cysteines inserted near the middle and C-terminus of the D4S6 segment alters channel gating and reduces Na^+^ current amplitude.

## Materials and Methods

### Cell Culture

tsA201 cells were grown in DMEM (Dubelcco’s modified Eagle’s medium) supplemented with 10% FBS, L-glutamine (2 mM), penicillin G (100 U/ml) and streptomycin (10 mg/ml) (Gibco BRL Life Technologies, Burlington, ON, Canada) in a 5% CO_2_ incubator. The cells were transfected using a standard calcium phosphate precipitation method (Invitrogen Corporation, Carlsbad, CA, USA). In order to facilitate the identification of transfected cells, 1 μg of CD8 plasmid was cotransfected with 1 μg of either WT or mutant Na^+^ channel cDNA and incubated 24–48 h. Immediately before recording the cells were incubated for 2 min with CD8 antibody-coated beads (Dynabeads, Invitrogen Corporation, Carlsbad, CA, USA) to label successfully transfected cells expressing the CD8 antigen.

### Electrophysiology

Whole-cell Na^+^ currents of tsA201 cells expressing Na_v_1.5 channels were obtained using an Axopatch 200A patch clamp amplifier equipped with a DigiData 1440A interface (Axon Instruments). Voltage pulses were generated and data collected using pClamp Version 9.0 (Axon Instruments). Patch pipettes fashioned from Corning 8161 glass (Warner Instruments, Hamden, CT, USA) had resistances of 0.5–1 MΩ, and were coated with sylgard to reduce capacitive transients. Extracellular solution contained (in mM): 145 NaCl, 2 KCl, 1.5 CaCl_2_, 1 MgCl_2_, and 10 HEPES pH 7.4 with NaOH. Internal solution consisted of (in mM): 105 CsF, 35 mM NaCl, 5 EGTA, and 10 Cs-HEPES pH 7.4 with CsOH. Thiol reactive MTSET [2-(Trimethylammonium) ethyl] methanethiosulfonate Bromide) was obtained from Toronto Research Chemicals (Toronto, ON, Canada). A stock solution of MTSET (100 mM) was made in distilled water and stored on ice for less than two hours. MTSET was diluted into internal solution to a final concentration of 1 mM immediately prior to filling the patch pipette. The time between pipette filling and establishing the whole-cell configuration was about 2 min. Peak amplitudes of the unmodified Na^+^ currents were determined from measurements made within the first 10 s of establishing the whole-cell configuration. Experiments in which break-in induced leak or in which unstable peak currents were observed were discarded. In these studies a cysteine situated near external mouth of the channel (C373) of Na_v_1.5 was replaced with phenylalanine (C373F) to prevent MTSET modification at this site.

### Molecular Biology

Amino acid substitutions were made using the QuickChange Site-Directed Mutagenesis Kit according to the supplier’s instructions (Stratagene Inc., La Jolla, CA, USA). Mutations substitutions were confirmed by cDNA sequencing.

### Data Analysis

The data are reported as the mean and SEM. The current amplitudes of the reagent-free control and MTSET-treated cells were compared using *t*-tests (*p* < 0.05).

### Model of Closed-State Inactivation

A first-order reaction was used to model the interconversion between resting and inactivated states. β and α are the voltage dependent rate constants for entering and exiting the inactivated state, respectively. The time constants of closed-state inactivation (τ_c_) were fitted to τ = (β + α)^-1^, where β(*V*) = β(0)exp(*V/k*) and α(*V*) = α(0)exp(–*V/k*). β(0) and α(0) are the rate constants at 0 mV, *V* is the test pulse voltage and *k* represents the voltage dependence. In this model the steady-state probability of not being inactivated is α/(β + α).

### Steady-State Inactivation

Steady-state inactivation was measured by applying 200 ms prepulses to voltages between –170 and –50 mV. A standard test pulse to –10 mV for 20 ms was used to assess current availability. The test currents were normalized to those measured after prepulsing to –170 mV and plotted versus the prepulse voltage. The data were fit to a Boltzmann function: (*I/I*_o_ = 1/[1–exp(*V*_0.5_–*V*)/*k*_v_]), where *I* is peak test current, I_o_ is the maximal current measured after prepulsing to –170 mV, V_0.5_ is the midpoint of inactivation and *k*_v_ the slope factor reflecting the voltage-sensitivity of inactivation.

### Recovery from Inactivation

Depolarizing prepulses to –10 mV for 20 ms were used to inactivate the channels before returning the membrane potential to –140 mV for variable intervals (0.1–250 ms). A standard test pulse to –10 mV for 20 ms was used to assess channel availability. The test currents were normalized to the currents measured after 250 ms at –140 mV and plotted versus the recovery interval. The smooth lines are biexponential curve fits [*I/I*_o_ = *A*_fast_(1–exp–*t*/τ_fast_) + (1–*A*_fast_)(1–exp(–*t*/τ_slow_)] where *I* is the amplitude of the test current, *I*_o_ the amplitude of the current measured after the 250 ms recovery interval, τ_fast_ and τ_slow_ the fast and slow recovery time constants and *A*_fast_ the relative amplitude of the fast component (Note:*A*_slow_ = 1–*A*_fast_).

### Na_v_1.5 Homology Model

Homology modeling of the Na_v_1.5 pore-forming region was based on the x-ray crystal structure of the Na_v_Ms bacterial Na^+^ channel. Model shows the predicted orientation of the Na_v_1.5 residues V1763 (purple), Y1767 (orange), and I1770 (cyan). Details of the modeling are further described in Poulin et al. (2014).

## Results

**FIGURE 1:**
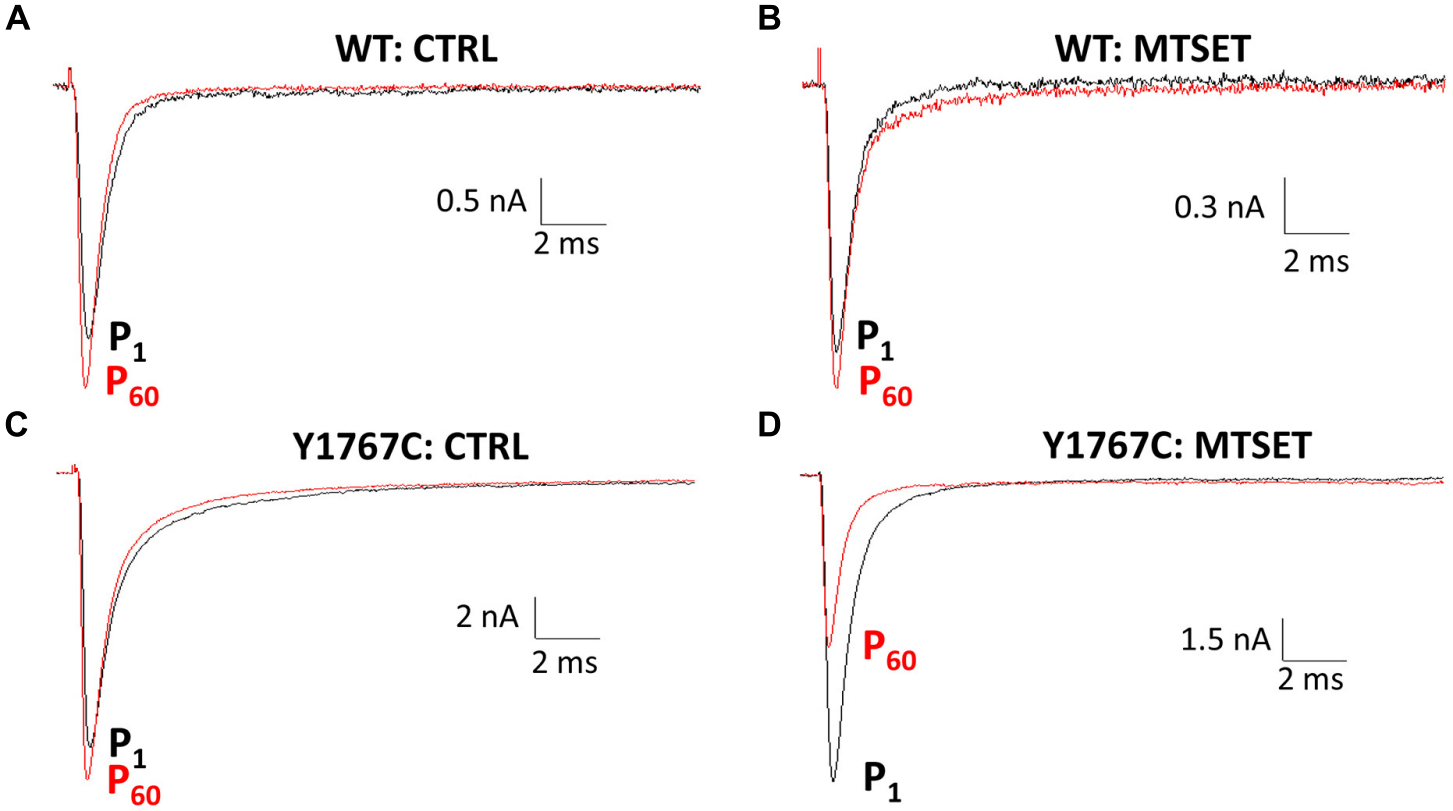
**Methanethiosulfonate reagent (MTSET) inhibition of D4S6 cysteine mutant**. Cells were held at –140 mV and depolarized to –10 mV for 20 ms at 5 s intervals. Currents are shown immediately after whole-cell break-in (P_1_) and after 5 min (P_60_). **(A,C)** In the absence of reagent the currents increased 29.1 ± 3.6% (*n* = 15) for wild-type and 21.6 ± 2.6% (*n* = 9) for Y1767C after 5 min. **(B)** Including 1 mM MTSET in the patch pipette had no effect on wild-type currents. **(D)** Internal MTSET inhibited the Y1767C currents by 42.1 ± 4.6% after 5 min.

**FIGURE 2:**
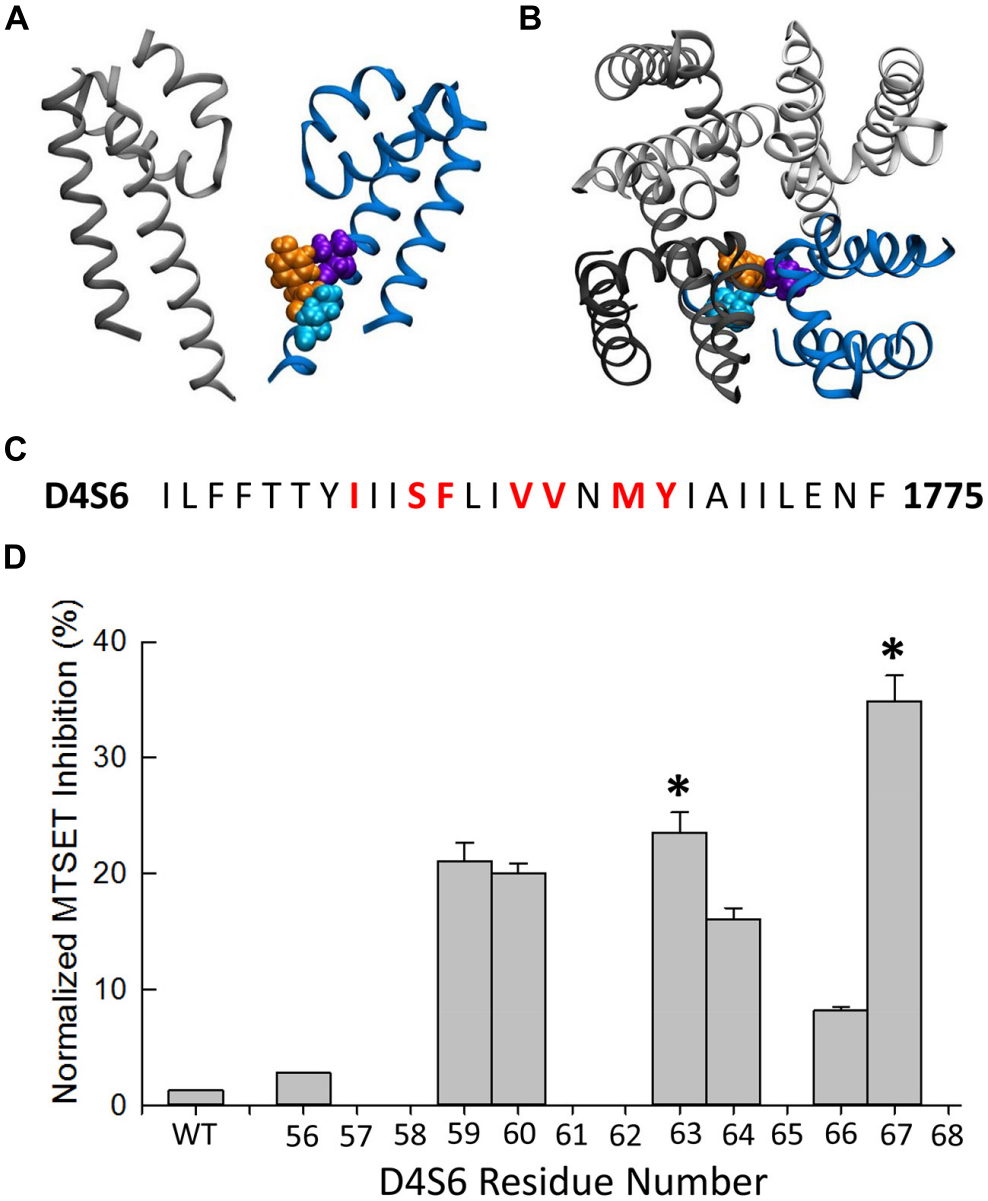
**Methanethiosulfonate reagent inhibition of D4S6 cysteine mutants. (A,B)** Homology model of the Na_v_1.5 pore-forming region (See Materials and Methods). Model shows the orientation of the Na_v_1.5 D4S6 segment (blue) residues V1763 (purple), Y1767 (orange), and I1770 (cyan) viewed from the side **(A)** and top **(B)** of the channel. The D2 segment is shown in gray and the D1 (front) and D3 (back) segments were removed for clarity. **(C)** Primary amino acid sequence of the Na_v_1.5 D4S6 segment. Residues mutated to cysteine are shown in red. **(D)** The Na^+^ currents of D4S6 cysteine mutants were measured immediately after break in (P_1_) and after 5 min of internal 1 mM MTSET (P_60_). MTSET inhibition was calculated from the ratio of the P_60_ and P_1_ currents [1–(I_P60_/I_P1_)^∗^100] and plotted versus the D4S6 residue number (I1756–Y1767). Asterisks indicate significant differences in the MTSET inhibition of the Na^+^ currents of adjacent pairs of D4S6 cysteine mutants.

Using a homology model based on bacterial Na^+^ channels as a guide (**Figure 2**), residues of the Na_v_1.5 D4S6 segment predicted to be exposed within the cytoplasmic pore were replaced with cysteines and the mutant channels heterologously expressed in tsA201 cells. **Figure 1** compares the effects of internally applied MTSET (1 mM) on the Na^+^ currents of wild-type Na_v_1.5 and the Y1767C cysteine mutant. In the absence of MTSET the peak currents slowly increased after break-in due to a combination of recovery from inactivation and intracellular dialysis of the cells with pipette solution (**Figures 1A,C**). In the absence of reagent, the peak currents of wild-type and Y1767C channels increased an average of 29.1 ± 3.6% (*n* = 15) and 21.6 ± 2.6% (*n* = 9), respectively. Including MTSET in the patch pipette resulted in increased wild-type currents similar to reagent-free controls (**Figure 1B**) indicating that in the absence of inserted D4S6 cysteines the Na_v_1.5 channels are insensitive to internally applied MTSET (O’Reilly and Shockett, 2012). This contrasted with the Y1767C mutant where internal MTSET produced a 42.1 ± 4.6% decrease in the Na^+^ current amplitude (**Figure 1D**).

The MTSET inhibition of the D4S6 cysteine mutants was quantified by calculating the ratio of the currents (P_60_/P_1_) measured immediately after break-in (P_1_) and after 5 min of internal MTSET exposure (P_60_). MTSET significantly inhibited the Na^+^ currents of the F1760C, V1763C, and Y1767C mutants (**Figure 2D**). We also examined the effects of MTSET on the currents of cysteine mutants situated immediately adjacent to these sites (S1759C, V1764C, M1766C). MTSET produced similar inhibition of the S1759C/F1760C and V1763C/V1764C pairs indicating that these adjacent cysteines are equally sensitive to internal MTSET. The MTSET inhibition of Y1767C was significantly larger than the adjacent M1766C suggesting that the latter residue may face away from the aqueous pore making it less accessible to polar MTSET.

### MTSET Produces Hyperpolarizing Shifts in Steady-State Inactivation

**FIGURE 3:**
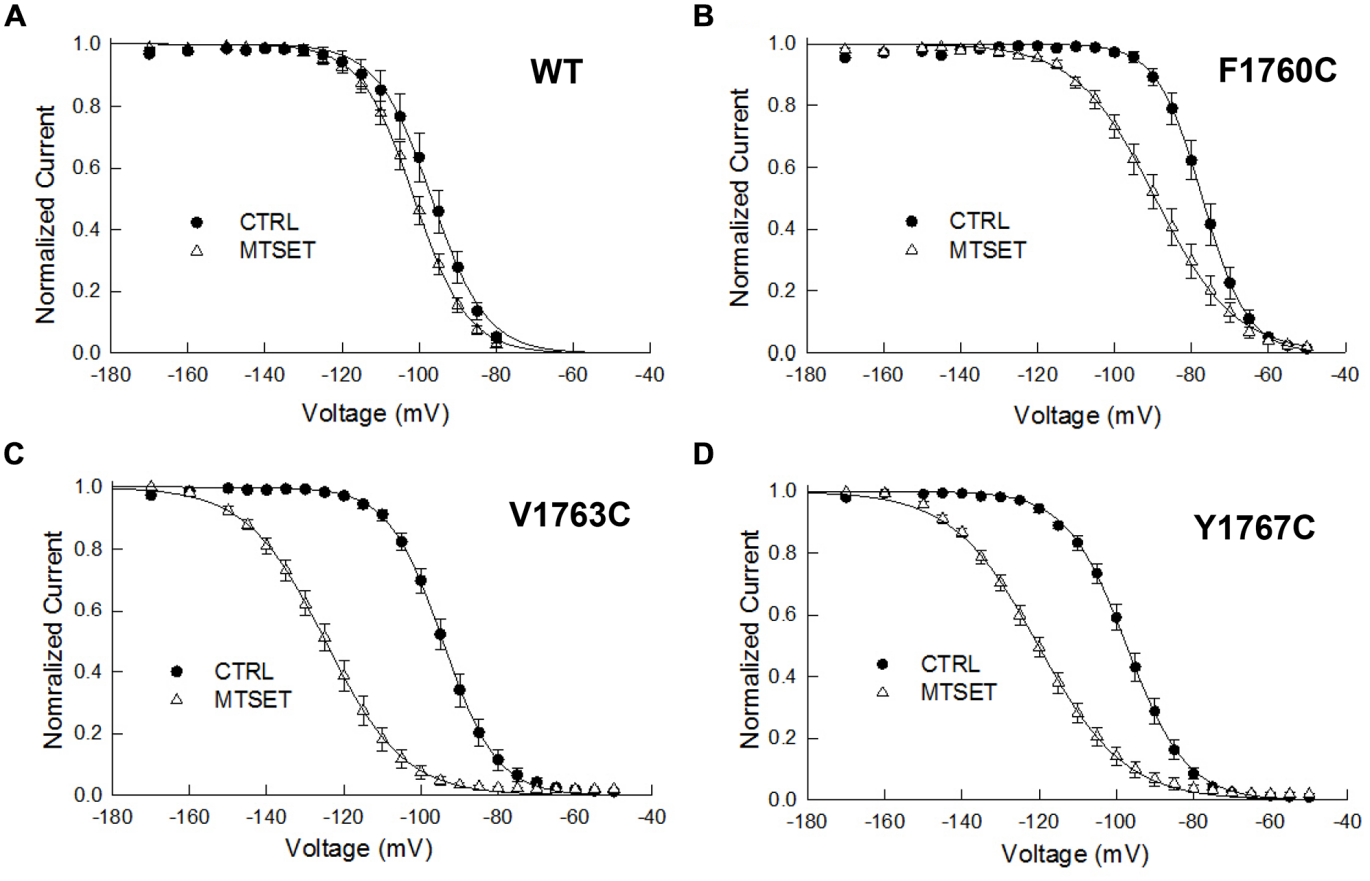
**Methanethiosulfonate reagent induces hyperpolarizing shifts in steady-state inactivation of D4S6 cysteine mutants**. Steady-state inactivation was determined using 200 ms prepulses to voltages between –170 and –50 mV and a standard test pulse to –10 mV for 20 ms to assess availability. The test pulses were normalized to the current measured at –170 mV and plotted versus prepulse potential. Data shown are for wild-type **(A)**, F1760C mutant **(B)**, V1763C mutant **(C)**, and Y1767C mutant **(D)** channels. The smooth curves are fits to Boltzmann functions with the midpoints (V0.5) and slope factors (*k*_v_) listed in **Table 1**.

The MTSET-induced reduction in the currents of the cysteine mutants could result from decreased availability caused by hyperpolarizing shifts in steady-state inactivation. Steady-state inactivation was determined using 200 ms prepulses to voltages between –160 and –50 mV. **Figure 3** plots the steady-state availability of the currents versus the prepulse potential. The inactivation of wild-type channels had a midpoint (V_0.5_) of –97 mV and slope factor (*k*_v_) of 5.7 mV. Internal MTSET produced a slight hyperpolarizing shift in V_0.5_ (ΔV_0.5_ = –3.8 mV) but no change in *k*_v_. In the absence of reagent the midpoints and slope factors of the V1763C and Y1767C mutants were similar to the wild-type channels indicating that the cysteine substitutions at these positions do not substantially alter inactivation. Internal MTSET shifted the midpoints of V1763C and Y1767C inactivation (ΔV_0.5_) by –31 and –23 mV, respectively and produced a 1.5-fold increase in *k*_v_ (**Table 1**).

**Table 1:** Biophysical properties of D4S6 mutants.

		Inactivation			Recovery			
		V_0.5_ (mV)	*k*_v_ (mV)	*n*	τ_fast_ (ms)	τ_slow_ (ms)	*A*_fast_	*n*
WT	CTRL	–97 ± 2.4	5.7 ± 0.4	12	2.7 ± 0.1	82 ± 11	0.95	11
	MTSET	–101.4 ± 1.3	6.6 ± 0.3	10	–	–	–	–
F1760C	CTRL	–77.2 ± 1.6	5.3 ± 0.1	8	1.0 ± 0.01	33 ± 9	0.92	8
	MTSET	–89.4 ± 2.1	9.2 ± 0.4	13	1.5 ± 0.1 †	139 ± 65 †	0.96	13
V1763C	CTRL	–94.0 ± 1.6	6.6 ± 0.2	8	3.6 ± 0.2	78 ± 12	0.97	7
	MTSET	–125.1 ± 2.0	9.3 ± 0.2	8	13.0 ± 0.8 †	– ^∗^	1.00	8
Y1767C	CTRL	–97.3 ± 1.3	7.5 ± 0.1	7	2.1 ± 0.2	52 ± 14	0.93	7
	MTSET	–120.3 ± 1.8	10.5 ± 0.4	10	4.5 ± 0.4 †	44 ± 13	0.89	10
I1770C	CTRL	–93.5 ± 0.1	6.5 ± 0.1	7	1.7 ± 0.1	24 ± 4	0.96	11

*† significant difference in pooled t-test (p < 0.05).*

*∗Recovery of MTSET-treated V1763C mutant was best fitted by a single exponential.*

**FIGURE 4:**
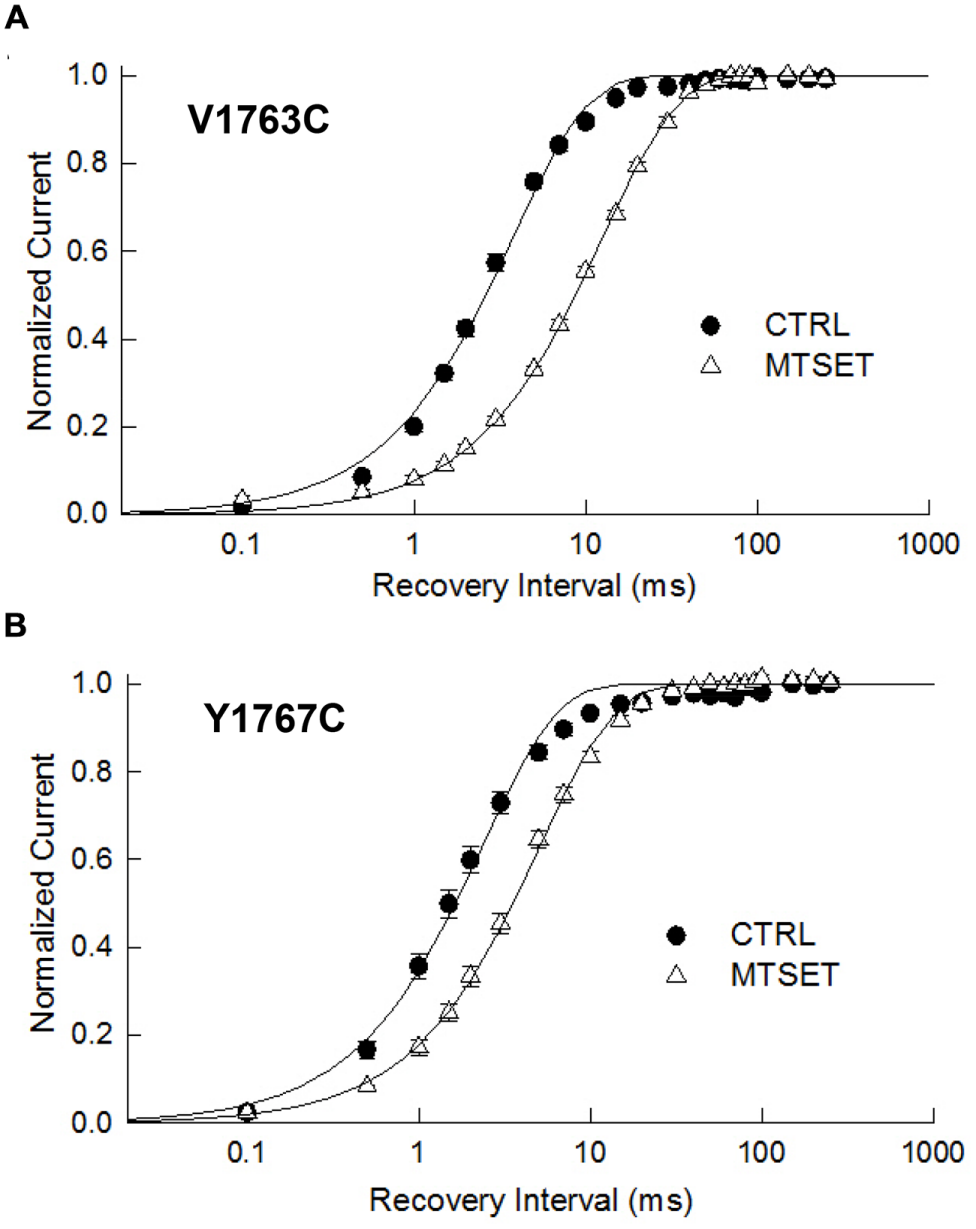
**Methanethiosulfonate reagent slows recovery from inactivation**. Depolarizing prepulses to –10 mV for 20 ms were used to the V1763C **(A)** and Y1767C **(B)** mutant channels. Cells were returned to –140 mV for a variable interval (0.1 ms–250 ms) before applying a standard test pulse to –10 mV for 20 ms. The test currents were normalized to the recovery measured after 250 ms of recovery and plotted versus the recovery interval. The smooth lines are biexponential curve fits with time constants and relative amplitudes of the fast components listed in **Table 1**.

The observed hyperpolarizing shifts in V1763C and Y1767C steady-state inactivation could result from MTSET-induced slowing of recovery from inactivation. In the absence of reagent the recovery time course of the V1763C and Y1767C mutants were exponential with time constants (τ_R_) of 3.8 and 2.4 ms respectively (**Figure 4**). MTSET delayed recovery and increased the recovery time constants of both V1763C (12.6 ms) and Y1767C (5.1 ms). The significant 2–3 fold increase in τ_R_ accounted for the slower recovery of the MTSET-modified channels and contributes to the observed hyperpolarizing shifts in steady-state inactivation (**Table 1**).

### Fast Inactivation Promotes MTSET Inhibition

**FIGURE 5:**
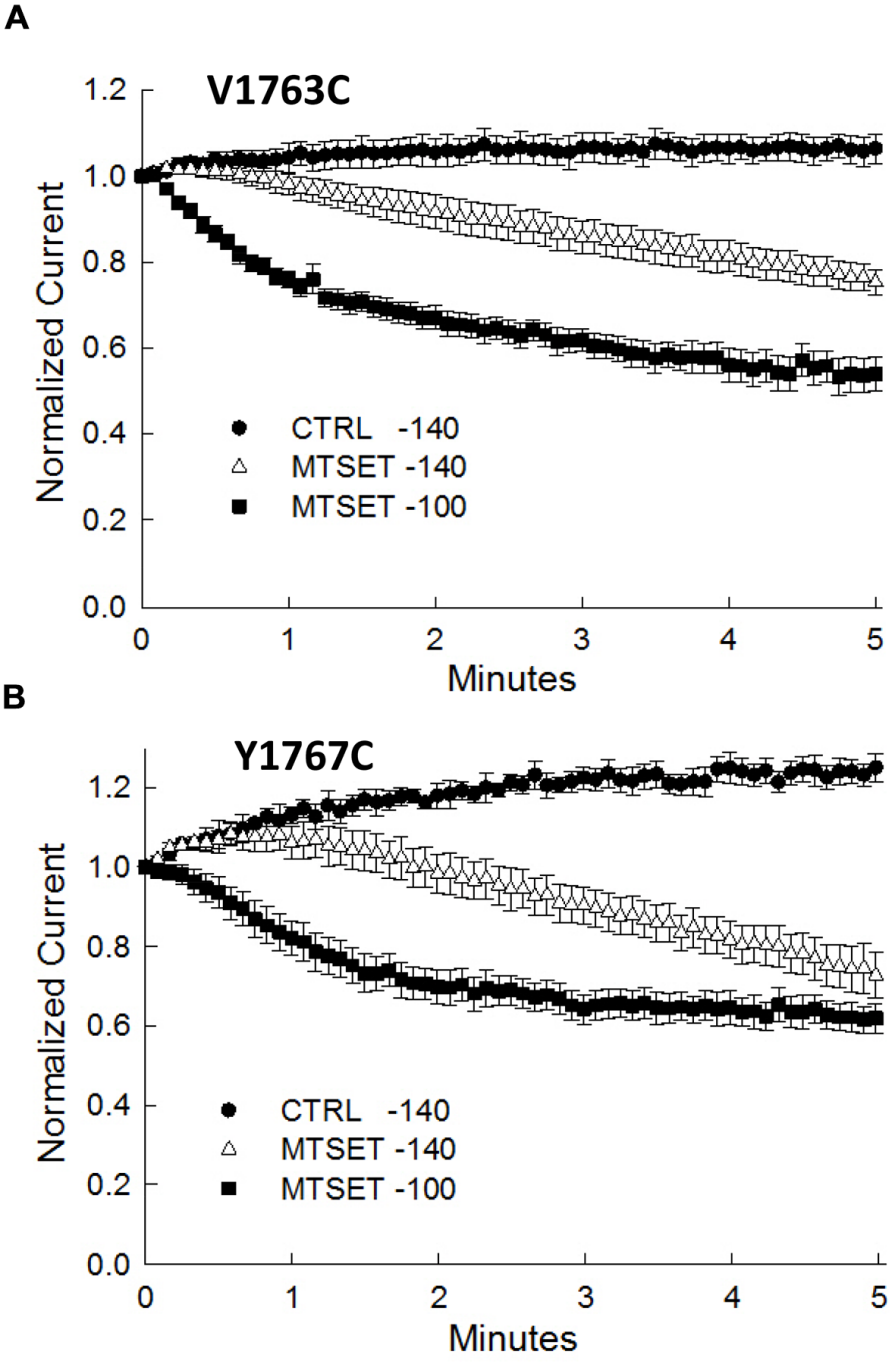
**Methanethiosulfonate reagent inhibition is voltage-dependent inhibition**. The MTSET inhibition of the V1763C **(A)** and Y1767C **(B)** mutants was examined while varying the holding potential between –140 and –100 mV. Standard test pulses to –10 mV for 20 ms were applied at 5 s intervals to assess current amplitude. The test currents were normalized to the current measured immediately after break-in. Plotted are the normalized currents for the V1763C and Y1767C mutants in the absence and presence of 1 mM internal MTSET. Holding at a more depolarized voltage (–100 mV) accelerated the onset of the MTSET inhibition by comparison to the –140 mV holding potential. The data are the means and SEM of the V1763C Control (*n* = 9), V1763C MTSET (*n* = 4), Y1767C control (*n* = 9), and Y1767C MTSET (*n* = 14).

During the course of these experiments we observed that depolarizing holding potentials (<–140 mV) appeared to facilitate the onset of MTSET inhibition of the D4S6 cysteine mutants (**Figure 5**). At –100 mV, the currents of the V1763C and Y1767C mutants were completely inhibited in ≈2 min of internal MTSET exposure. This contrasted with the –140 mV holding potential where the MTSET inhibition required >5 min to reach steady-state levels. Steady-state inactivation predicts that the fraction of inactivated channels at –140 mV (V1763C: 0.9 ± 0.003%, Y1767C: 2.2 ± 0.02%) significantly increases at –100 mV (V1763C: 30.2 ± 1.7%, Y1767C: 54.8 ± 9.6%) for both mutants. These data suggest that holding the channels at more depolarized voltages where a larger fraction of the channels are inactivated accelerates the onset of the MTSET inhibition.

### Fast Inactivation is Required for MTSET Inhibition

**FIGURE 6:**
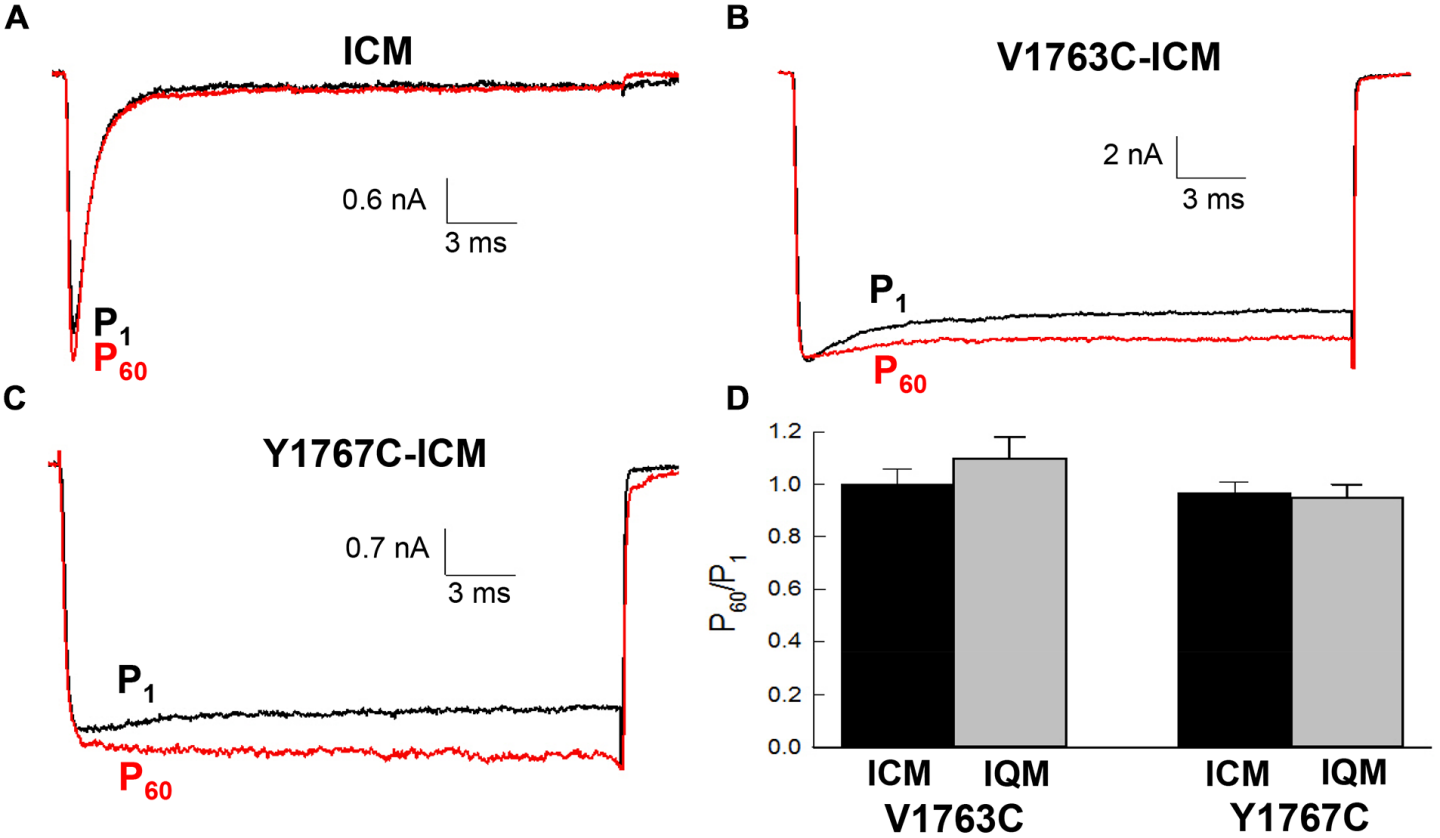
**Methanethiosulfonate reagent inhibition requires fast inactivation**. The V1763C and Y1767C mutations were transferred to an inactivation deficient background of Na_v_1.5 created by replacing the conserved F1486 of the interdomain D3–D4 linker with either cysteine or glutamine (see text). **(A)** Shows the current of the ICM mutant lacking D4S6 mutations measured during perfusion with standard pipette solution. **(B)** Current of the V1767C-ICM mutant measured with 1 mM MTSET in the patch pipette. **(C)** Current of the Y1767C-ICM mutant measured in the presence of MTSET. **(D)** Currents were measured immediately after break in (P_1_) and after 5 min (P_60_). The P_60_/P_1_ ratios of the V1763C-ICM and Y1767C-ICM mutants were not different after 5 min of internal MTSET. Also shown are the P_60_/P_1_ ratios of the non-inactivating V1763C-IQM and Y1767C-IQM mutants.

The contribution of fast inactivation to MTSET inactivation was further investigated by transferring the V1763C and Y1767C mutations to an inactivation-deficient background of Na_v_1.5 constructed by substituting cysteine for the native phenylalanine (F1486C) of the D3–D4 linker IFM motif (Kellenberger et al., 1996; Chahine et al., 1997). Internal MTSET has been shown to rapidly modify the cysteine at position 1486 resulting is the loss of fast inactivation. Internally applied MTSET abolished the fast inactivation of the V1763C-ICM and Y1767C-ICM mutant channels within seconds (<10 s) of break-in indicating that MTSET rapidly diffuses from the pipette into the cell (**Figures 6B,C**). Despite the presence of internal MTSET the peak currents of the non-inactivating V1763C-ICM and Y1767C-ICM channels gradually increased in amplitude similar to reagent-free controls. **Figure 6D** shows that after 5 min of internal exposure the normalized currents of MTSET-treated cells were not different from reagent-free controls.

Also shown is the effect of internal MTSET on an alternative non-inactivating Na_v_1.5 background (IQM) constructed by replacing the phenylalanine of the IFM motif with glutamine (F1486Q). In the absence of MTSET the inactivation of the V1763C-IQM and Y1767C-IQM double mutants were severely impaired resulting in channels that remain persistently open at depolarized voltages (West Patton Scheuer Wang Goldin Catterall 1992). Internal MTSET did not inhibit the currents of the V1763C-IQM or Y1767C-IQM channels (**Figure 6D**).

### Mutations Near the C-Terminus of the D4S6 Disrupt Fast Inactivation

Situated near the C-terminus of the D4S6 segment is an isoleucine (I1770) that bacterial models predict is located near the narrow region of the cytoplasmic pore. Replacing this isoleucine with cysteine (I1770C) produced a small but significant increase in the persistent current (1.8 ± 0.1%, *n* = 7) by comparison to wild-type channels (0.5 ± 0.04%, *n* = 13, *t*-test, *p* < 0.05). I1770C also induced a slight depolarizing shift in steady-state inactivation (ΔV_0.5_ = 3.5 mV) and accelerated the recovery from inactivation (**Table 1**). The I1770C mutation mildly disrupts fast inactivation.

**FIGURE 7:**
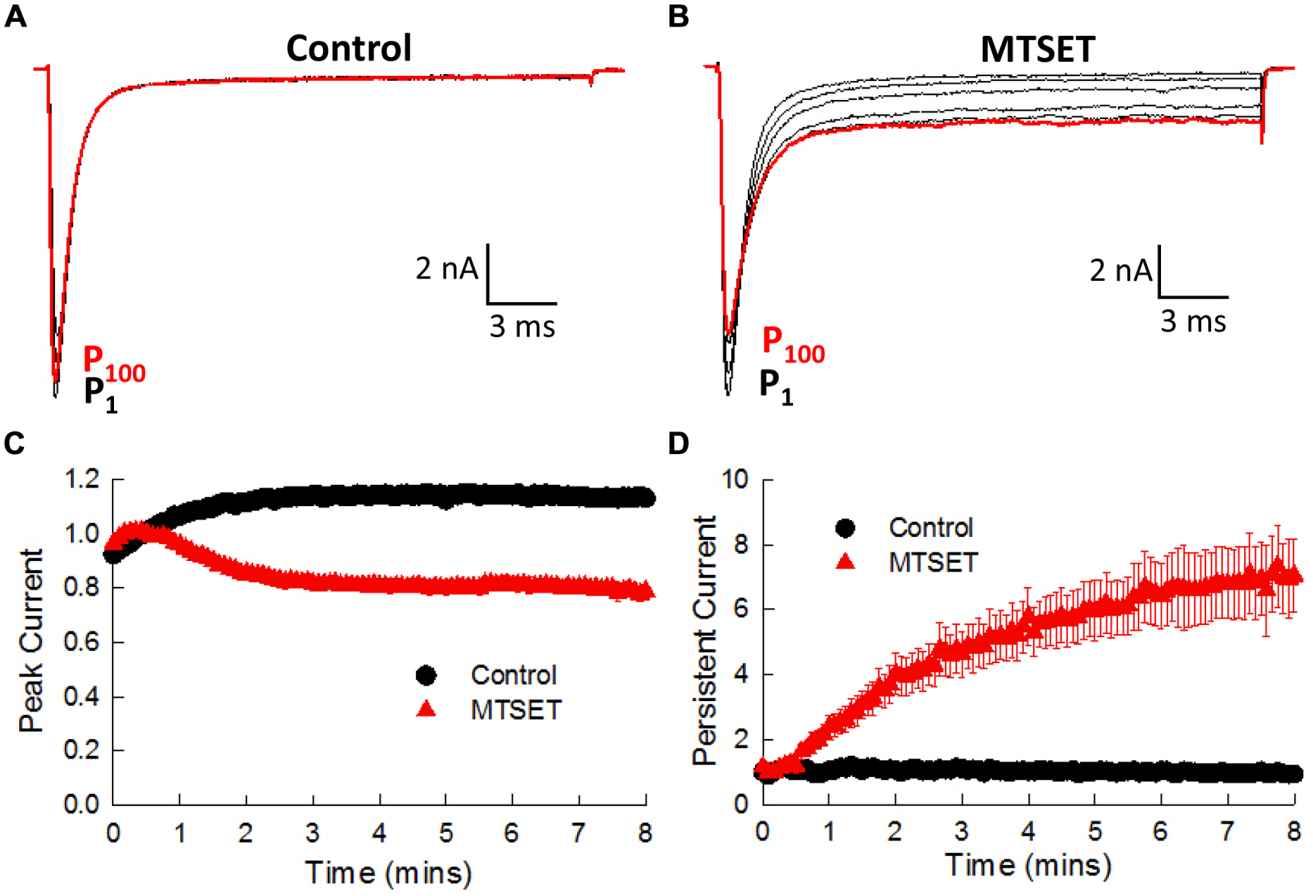
**Internal MTSET induces a persistent current in the I1770C mutant. (A)** The currents of the I1770C mutant measured in reagent-free pipette solution. Currents are shown immediately after break-in (P_1_) and after 0.5, 1, 3, 5, and 8 min (P_100_). **(B)** Currents of the I1770C mutant with 1 mM MTSET in the patch pipette. **(C)** Peak currents measured in the absence and presence of MTSET. **(D)** Persistent current measured after 25 ms of depolarization.

In the absence of MTSET the I1770C currents gradually increased (19.5 ± 1.0%, *n* = 4) during the first 3 min and thereafter remained constant (**Figure 7A**). Including MTSET in the patch pipette reduced the I1770C peak currents and increased the residual non-inactivating current measured after 25 ms of depolarization (**Figure 7B**). The peak and persistent currents were normalized to the currents measured immediately after break-in (P_1_) and plotted versus the duration of MTSET exposure (**Figures 7C,D**). After 5 min, internal MTSET significantly reduced the peak currents 32.0 ± 2.1% and increased the persistent current sixfold by comparison to reagent-free controls (*t*-test, *p* < 0.05). The decrease in I1770C peak current was similar to the MTSET inhibition of the V1763C (23.7%) and Y1767C (42.1%) mutants. The MTSET-induced increase in I1770C persistent current was not observed with the other D4S6 mutants. Both the direct effects of the I1770C mutation on steady-state inactivation and recovery along with the observed increase in persistent current produced by internal MTSET suggest that residues situated near the C-terminus of the D4D6 may play a unique role in fast inactivation.

### F1760C Impairs Fast Inactivation

**FIGURE 8:**
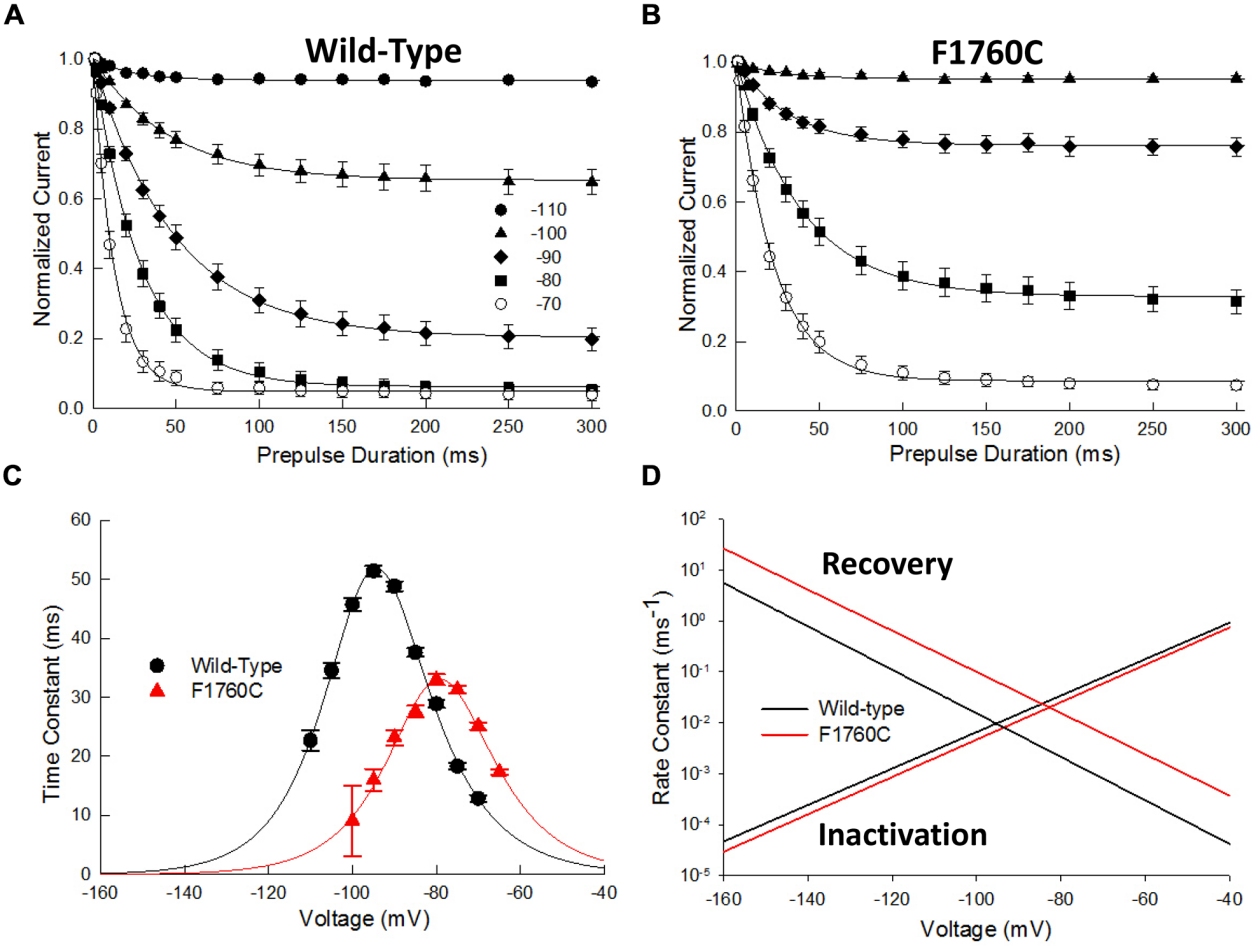
**The F1760C mutant alters closed-state inactivation**. The onset of closed-state inactivation was measured by applying variable length prepulses (0–150 ms) to voltages between –110 and –70 mV. The cells were then returned to –140 mV for 1 ms before assessing availability with a standard test pulse to –10 mV for 20 ms. The peak currents were normalized and plotted versus the prepulse pulse duration. **(A,B)** Onset of closed-state inactivation of the wild-type and F1760C channels. The smooth curves are fits to single exponential functions. **(C)** Time constants of the wild-type and F1760C mutant obtained from panels **(A,B)** plotted versus the prepulse potential. The smooth curve is a fit to a two state model yielding the inactivation (β) and recovery (α) rate constants (See Materials and Methods). **(D)** Plot of β and α as a function of voltage.

While the majority of the D4S6 cysteine mutations did not directly alter steady-state inactivation, F1760C produced a significant 20 mV depolarizing shift in inactivation midpoint (**Figure 3B**; **Table 1**). The underlying cause of this shift was investigated by measuring the time course of closed-state inactivation of the F1760C mutant at voltages between –110 and –70 mV. The development of closed-state inactivation at each voltage was with a fitted single exponential (**Figures 8A,B**) and the time constants plotted versus the prepulse voltage (**Figure 8**). The resulting bell-shaped curves were fitted to a two-state model yielding estimates of the inactivation (k_α_) and recovery (k_β_) rate constants (Material and Methods). The inactivation rate constants of the wild-type (β(0) = 24.7 ms^-1^) and F1760C (β(0) = 12.7 ms^-1^) displayed modest two-fold difference (**Figure 8D**). This contrasted with the recovery rate constant of the F1760C mutant (α(0) = 9.1 × 10^-6^ ms^-1^), which was an order of magnitude larger than recovery rate constant of wild-type channels (α(0) = 8.3 × 10^-7^ ms^-1^). The midpoint of F1760C steady-state inactivation (V_0.5_) predicted from β and α was –79 mV, similar to the value obtained using a standard double-pulse protocols (**Figure 3B**, V_0.5_ = –77 mV). These data indicate that the depolarizing shift in steady-state inactivation observed with the F1760C mutant is primarily driven by rapid recovery from inactivation with little or change in the onset of fast inactivation. Recent modeling suggests that T206 of the Na_v_Ms channel (S1759 in Na_v_1.5) is situated within a flexible region of the D4S6 and may contribute to a hinge that enables the C-terminus of the D4S6 to swing outward during channel opening (McCusker et al., 2012). Mutating residues within this region could alter hinge flexibility and may underlie the observed changes in F1760C steady-state inactivation.

Internal MTSET produced a 19 ± 5% decrease in F1760C current. Cysteine substitution at the adjacent position (S1759C) produced a slightly larger reduction (25 ± 5%) indicating that the cysteines at positions 1759 and 1760 are sensitive to MTSET. Unlike other D4S6 cysteine mutations, the F1760C-ICM channels were reduced 10.2 ± 4.5% by MTSET suggesting that the residue at this position remains accessible under non-inactivating conditions. In addition to inhibiting the F1760C current, internal MTSET induced a hyperpolarizing shift in steady-state availability (ΔV_0.5_ = –12.2 mV), a 1.7-fold increase in *k*_v_ and 1.5-fold slower recovery from inactivation (**Table 1**). Overall, the effects of internal MTSET on F1760C were relatively weak by comparison to those observed for the V1763C and Y1767C mutants.

## Discussion

The goal of this study was to investigate the role of the D4S6 segment in the gating of Na_v_1.5 channels. Guided by structural models of prokaryotic Na^+^ channels, D4S6 cysteines were introduced at positions predicted to be exposed within the cytoplasmic aqueous pore. Both the direct effects of the cysteine mutations and internal MTSET reagent on channel gating were examined. With the exception of F1760C, the D4S6 cysteine mutations were well tolerated and produced only minor changes in Na^+^ channel gating. Including MTSET in the patch pipette reduced the current amplitudes, slowed recovery from inactivation and produced hyperpolarizing shifts in steady-state inactivation of channels with cysteines located near the near the middle (V1763C) and C-terminus (Y1767C) of the D4S6. Internal MTSET had no effect on wild-type channels indicating that the observed effects do not result from the modification of endogenous cysteines. The MTSET-induced decrease in current amplitude is primarily caused by slow recovery from inactivation and the associated reduction in steady-state availability at negative holding potentials (–140 mV). This is supported by data showing that more depolarized holding potentials (–100 mV) that favor inactivation accelerated the onset of the MTSET-induced reduction in current (**Figure 5**). Overall, the findings indicate that inactivated channels are more susceptible to MTSET modification and that once modified the channels recover more slowly from inactivation.

The effects of MTSET are consistent with an important role for V1763 and Y1767 in fast inactivation. This mechanism was investigated by transferring V1763C and Y1767C to non-inactivating Na_v_1.5 channels (ICM, IQM). Internal MTSET failed to reduce the Na^+^ currents when the D4S6 cysteine mutations were co-expressed in these non-inactivating backgrounds (**Figure 6**). Because these channels remain persistently open at depolarized voltages suggests that the entry of polar MTSET into the cytoplasmic pore is not the rate limiting step in the MTSET inhibition. Rather the data further supports the idea that fast inactivation is required for the MTSET-induced reduction in Na^+^ current amplitude.

I1770 is located near the C-terminus of the D4S6 segment. In addition to reducing the peak currents of the I1770C mutant, MTSET also increased the residual current measured near the end of depolarizing pulses. MTSET did not alter the leak current measured when the channels were returned to their holding potential of –140 mV indicating that the I1770C channels close normally at hyperpolarized voltages. Rather the observed increase in residual current appears to be specific for the activated or inactivated states of the channel. We hypothesize that MTSET modification of the cysteine at this position 1770 enables the mutant channels to re-open from the normally absorbing inactivated state. This was unique to I1770C and was not observed with the other D4S6 mutations. I1770 is located near a proposed narrow region of prokaryotic Na^+^ channel pore and five residues upstream from the putative activation gate (Bagneris et al., 2014). Our data suggests that residues within this region may play a specific role in Na^+^ channel inactivation.

Methanethiosulfonate reagent is a positively charged reagent that covalently modifies cysteine residues exposed within aqueous environments (Vedantham and Cannon, 2000). Residues located within hydrophobic environments, such as those that are oriented away from the aqueous pore or those buried at protein interfaces, are generally insensitive to polar MTSET. Our findings are consistent with a mechanism in which the internally applied MTSET covalently modifies the V1763C and Y1767C cysteines resulting in slower recovery from inactivation, hyperpolarized shifts in steady-state inactivation and decreased availability of the channels. Moreover, the data show that MTSET modification is favored by depolarization suggesting that the cysteinyl side chains are more accessible when the channels are inactivated. The data indicate that the V1763C and Y1767C residues are preferentially exposed within cytoplasmic aqueous pore when the channels are inactivated. Modification of these residues with a cation adduct appears to stabilize these residues within the aqueous pore resulting slower recovery from inactivation. Y1767 is a well-known component of the Na_v_1.5 local anesthetic binding site (O’leary and Chahine, 2002; Huang et al., 2011). We speculate that exposure of Y1767 within the cytoplasmic pore of inactivated channels may contribute to the state-dependent binding of local anesthetics while drug binding to its aromatic sidechain may stabilize the aqueous exposure of this residue resulting in slower recovery from inactivation.

## Conflict of Interest Statement

The authors declare that the research was conducted in the absence of any commercial or financial relationships that could be construed as a potential conflict of interest.
